# Age-Dependent Effects of UCP2 Deficiency on Experimental Acute Pancreatitis in Mice

**DOI:** 10.1371/journal.pone.0094494

**Published:** 2014-04-10

**Authors:** Sarah Müller, Hannah Kaiser, Burkhard Krüger, Brit Fitzner, Falko Lange, Cristin N. Bock, Horst Nizze, Saleh M. Ibrahim, Georg Fuellen, Olaf Wolkenhauer, Robert Jaster

**Affiliations:** 1 Division of Gastroenterology, Department of Medicine II, University Medicine Rostock, Rostock, Germany; 2 Division of Medical Biology, University Medicine Rostock, Rostock, Germany; 3 Department of Neurology, University Medicine Rostock, Rostock, Germany; 4 Oscar-Langendorff-Institute of Physiology, University Medicine Rostock, Rostock, Germany; 5 Institute of Pathology, University Medicine Rostock, Rostock, Germany; 6 Department of Dermatology, University of Lübeck, Lübeck, Germany; 7 Institute for Biostatistics and Informatics in Medicine and Ageing Research, University Medicine Rostock, Rostock, Germany; 8 Department of Systems Biology and Bioinformatics, University of Rostock, Rostock, Germany; 9 Stellenbosch Institute for Advanced Study, Wallenberg Research Centre at Stellenbosch University, Stellenbosch, South Africa; Klinikum rechts der Isar der TU München, Germany

## Abstract

Reactive oxygen species (ROS) have been implicated in the pathogenesis of acute pancreatitis (AP) for many years but experimental evidence is still limited. Uncoupling protein 2 (UCP2)-deficient mice are an accepted model of age-related oxidative stress. Here, we have analysed how UCP2 deficiency affects the severity of experimental AP in young and older mice (3 and 12 months old, respectively) triggered by up to 7 injections of the secretagogue cerulein (50 μg/kg body weight) at hourly intervals. Disease severity was assessed at time points from 3 hours to 7 days based on pancreatic histopathology, serum levels of alpha-amylase, intrapancreatic trypsin activation and levels of myeloperoxidase (MPO) in lung and pancreatic tissue. Furthermore, *in vitro* studies with pancreatic acini were performed. At an age of 3 months, UCP2^-/-^ mice and wild-type (WT) C57BL/6 mice were virtually indistinguishable with respect to disease severity. In contrast, 12 months old UCP2^-/-^ mice developed a more severe pancreatic damage than WT mice at late time points after the induction of AP (24 h and 7 days, respectively), suggesting retarded regeneration. Furthermore, a higher peak level of alpha-amylase activity and gradually increased MPO levels in pancreatic and lung tissue were observed in UCP2^-/-^ mice. Interestingly, intrapancreatic trypsin activities (*in vivo* studies) and intraacinar trypsin and elastase activation in response to cerulein treatment (*in vitro* studies) were not enhanced but even diminished in the knockout strain. Finally, UCP2^-/-^ mice displayed a diminished ratio of reduced and oxidized glutathione in serum but no increased ROS levels in pancreatic acini. Together, our data indicate an aggravating effect of UCP2 deficiency on the severity of experimental AP in older but not in young mice. We suggest that increased severity of AP in 12 months old UCP2^-/-^ is caused by an imbalanced inflammatory response but is unrelated to acinar cell functions.

## Introduction

With an annual incidence of 13–45 cases per 100.000 persons, acute pancreatitis (AP) is among the leading causes of hospitalization for gastrointestinal disorders worldwide [1]. Given that mortality ranges from 3% for patients with edematous AP [2] to up to 30% in severe cases [1], treatment of the disease remains a clinical challenge. For decades, AP has been considered only as an autodigestive disease caused by intrapancreatic activation of digestive proteases, and indeed it is well established that premature trypsinogen activation leads to acinar cell death by enzymatic necrosis, which represents an important component of acinar injury in AP (reviewed in [3]). More recent studies have added activation of intense inflammatory signaling mechanisms in acinar cells to the key mechanisms of AP pathogenesis. Furthermore, observations about the occurrence of local and systemic inflammation in AP, independently of premature trypsinogen activation, have challenged solely trypsin-centered theories of the disease [3].

Reactive oxygen species (ROS) have been implicated in pancreatitis many years ago [4], [5], but still the precise role of ROS in the pathogenesis of the disease remains controversial. On one hand, increased ROS levels have been observed early in the course of AP, and preclinical studies have often suggested beneficial effects of antioxidant treatments [6]–[8]. Clinical studies with antioxidants, on the other hand, have yielded conflicting and frequently disappointing results [9]–[11]. Moreover, recent studies have put the simple concept of a general detrimental action of ROS into question by showing that increases of intracellular and mitochondrial ROS concentrations during bile acid injury of pancreatic acinar cells selectively promote apoptosis as a predominantly protective form of cell death in the context of AP [12].

Uncoupling protein 2 (UCP2) is a mitochondrial inner membrane carrier protein [13] that is expressed in many tissues, including pancreas [14]–[16] and specifically pancreatic acinar cells [16]. Accumulating evidence suggests that UCP2 functions as a negative regulator of mitochondria-derived ROS production by decreasing the mitochondrial membrane potential [17]–[19]. Thus, experimental overexpression of UCP2 provides cytoprotection by limiting ROS formation [20], [21], while defects of UCP2 expression or an inhibition of UCP2 function display the opposite effect [22], [23]. Interestingly, an increase of UCP2 expression in ageing tissues (liver and skeletal muscle) has been observed and may be important to attenuate ageing-associated oxidative stress burden, as also suggested by studies in UCP2^-/-^ mice [24], [25].

Deletion of UCP2 has been shown to severely affect immune responses, first of all but not exclusively by favoring macrophage activity (through increased ROS production and enhancement of ROS signaling; reviewed in [26]). Furthermore, an *ucp2* promoter polymorphism has been linked to chronic inflammatory diseases such as rheumatoid arthritis and systemic lupus erythematosus [27].

In two models of experimental AP, pancreatic UCP2 mRNA levels were found to be increased and correlated with the severity of the disease [16]. The authors proposed that up-regulation of UCP2 in the pancreas may be a protective response to oxidative stress, but also discussed a potential negative effect of UCP2 on cellular energy metabolism. Furthermore, they suggested acinar UCP2 as an important modifier of the severity of AP.

Here, we took advantage of the UCP2^-/-^ mouse model to study the role of ROS in the pathogenesis of experimental acute AP in a well-defined genetic context, and to directly assess specific functions of UCP2. Therefore, two age groups of mice, young adults (3 months old) and older animals (12 months old), were employed. Our data indicate that 12 months old UCP2^-/-^ mice but not the younger individuals are prone to develop a more severe disease than wild-type (WT) controls. Furthermore, the results provide insights into underlying cellular and molecular mechanisms.

## Materials and Methods

### Reagents

Unless stated otherwise, all reagents were obtained from Sigma-Aldrich (Deisenhofen, Germany).

### Animal Studies

UCP2^-/-^ mice [28] were purchased from Charles River Laboratories (Sulzfeld, Germany) and kept on the C57BL/6 background (WT control strain). All experiments were performed according to the guidelines of the local animal use and care committee, which also approved this study (Landesamt für Landwirtschaft, Lebensmittelsicherheit und Fischerei Mecklenburg-Vorpommern, permit number for the study: LALLF M-V/TSD/7221.3-1.1-077/11). The mice had access to water and standard laboratory chow ad libitum. All animals received humane care according to the German legislation on protection of animals and the Guide for the Care and Use of Laboratory Animals (NIH publication 86–23, revised 1985), and all efforts were made to minimize suffering.

The studies were performed with balanced numbers of female and male mice at an age of 3 or 12 months as indicated. Prior to the experiments, the mice were fasted overnight with free access to water. Afterwards, the secretagogue cerulein (Bachem, Heidelberg, Germany) was administered in seven intraperitoneal injections of 50 μg/kg body weight at hourly intervals [29], [30]. Mice were euthanized by an overdose of ketamine/xylazin hydrochloride at intervals between 3 hours and 7 days after the first intraperitoneal injection of cerulein. Lung tissue, pancreas and serum (obtained from whole blood) were harvested and stored under appropriate conditions (−80°C for shock-frozen native tissue) until they were assayed.

### Amylase Measurement

Alpha-amylase (α-amylase) activity in serum was determined in a routine laboratory using the IFCC reference method [31] (AMY reagent; Beckman Coulter, Mervue, Galway, Ireland).

For the detection of pancreatic α-amylase, ≥30 mg of pancreatic tissue was homogenized in phosphate-buffered saline (PBS; pH 7.0, 20 mmol/L) using an Ultra-Turrax T10 disperser (IKA works, Staufen, Germany) and subjected to sonication. All steps were performed on ice and with pre-cooled reagents and instruments. After clearing the lysates by centrifugation, the supernatants were stored at −80°C until assayed. Measurements of α-amylase activity were performed as described above.

### Histology, Immunohistochemistry and Detection of Apoptotic Cells *in situ*

For histology, pancreata were fixed in 4% formaldehyde phosphate buffer overnight and processed for paraffin embedding. Routine hematoxylin and eosin (H&E) staining for determination of organ inflammation was performed on 1 μm sections using standard procedures. Histopathological evaluation of pancreatic injury was performed by light microscopy. Severity of organ inflammation was determined on blinded samples, employing a semiquantitative score to assess the following four key parameters of cerulein-induced AP [29], [30]: (1) the degree of edema, (2) the presence of potentially reversible cellular damage (cell swelling, vacuolization and cystic degeneration), (3) the frequency of dead (apoptotic or necrotic) cells, and (4) the number of infiltrating inflammatory cells. Each parameter was evaluated on a scale from 0–3 (0 being normal and 3 being severe) and the sum was calculated, resulting in a maximum total score of 12.

Infiltrating inflammatory cells were further classified by immunohistochemical analysis, applying an avidin–biotin-peroxidase (ABC) technique and reagents that were delivered (unless indicated otherwise) by Vector Laboratories (Burlingame, CA, USA). Therefore, air-dried cryostat sections (6 μm) were fixed by incubation in ice-cold methanol for 30 s. After three washing steps with PBS, sections were treated, in two subsequent steps of 15 min, with Avidin D and biotin solution (Avidin/Biotin blocking kit) to block endogenous biotin and biotin-binding proteins. Next, slides were incubated for 60 min with PBS containing 2.0% goat serum, followed by exposure to rat anti-mouse antibodies against CD11b (ImmunoTools, Friesoythe, Germany), Gr1 (eBioscience, Frankfurt, Germany) and CD3 (BD Pharmingen, Heidelberg, Germany) diluted in 2.0% goat serum/PBS for 90 min. Subsequently, sections were washed and exposed to biotinylated goat anti-rat immunoglobulin (diluted in 2.0% goat serum/PBS) for 30 min. After further washing steps, the sections were incubated with ABC reagent for 30 min, before target proteins were visualized using the peroxidase substrate and chromogen *Nova*RED. Slides were counterstained with Mayer's hemalum solution, dehydrated by four short incubations in ethanol and xylene (two times each) and embedded in Pertex (MEDITE, Burgdorf, Germany).

Apoptotic cells in pancreatic tissue were detected employing the *ApopTag Peroxidase Apoptosis Detection Kit* according to the instructions of the manufacturer (Millipore, Billerica, MA, USA). The kit is based on the TUNEL method and stains apoptotic cells *in situ* by labeling DNA strand breaks. Briefly, cryostat sections (6 μm) were fixed by incubation in paraformaldehyde (1%) for 10 min at room temperature (RT). Next, the tissue was permeabilized by incubation in ethanol/acetic acid (2∶1 mixture) for 5 min at −20°C before endogenous peroxidase activity was blocked with hydrogen peroxide solution (3%; 5 min at RT). After short incubation with *Equilibration Buffer, Terminal Deoxynucleotidyl Transferase Enzyme* solution was applied to the tissue (60 min at 37°C), before the labeling reaction was stopped with *Stop/Wash Buffer*. Subsequently, *Anti-Digoxigenin Peroxidase Conjugate* was added (30 min at RT). Finally, tissue sections were incubated with the peroxidase substrate 3,3′ diaminobenzidine for 5 min, counterstained with methyl green at 0.5% (10 min), dehydrated with xylene and embedded in Pertex.

Positive-stained cells (ABC staining *ApopTag Kit, respectively*) were counted in ten representative areas per section (one area  = 0.09 mm^2^).

### Detection of Trypsin Activity *in vivo*

Pancreata were homogenized in MOPS buffer (5 mmol/L MOPS pH 6.5, 1 mmol/L MgSO_4_, 250 mmol/L Saccharose) and trypsin activity was determined fluorometrically following published protocols [32]. All steps were performed on ice and with pre-cooled reagents. To determine the content of active trypsin in the homogenate, the synthetic substrate BOC-Gln-Ala-Arg-7-amido-4-methylcoumarin HCl (Peptanova, Sandhausen, Germany) was applied at a final concentration of 30 μmol/L in Tris buffer (50 mmol/L Tris pH 8.0, 150 mmol/L NaCl, 1 mmol/L CaCl_2_, 0.01% BSA). Fluorescence of the cleavage product 7-amino-4-methylcoumarin (AMC) was recorded using an *Infinite 200 microplate reader* (Tecan Group Ltd., Männedorf, Switzerland) and an excitation/emission wavelength of 360/438 nm, respectively (reaction temperature: 30°C, 20 cycles of 30 s measurement). Protein concentrations of the homogenates were quantified according to Bradford [33]. Trypsin concentration was calculated using bovine trypsin as a standard.

### Measurement of Myeloperoxidase (MPO) Activity

MPO activities in pancreatic and lung tissue were determined using the MPO fluorometric detection kit from Enzo Life Sciences (Lörrach, Germany). The kit uses a non-fluorescent detection reagent, which is oxidized in the presence of hydrogen peroxide and MPO to produce its fluorescent analog. All steps were performed on ice and with pre-cooled reagents. Approximately 100 mg of lung and ≥30 mg of pancreatic tissue was homogenized in *assay buffer* supplemented with N-Ethylmaleimide (10 mmol/L). Samples of lung tissue were adjusted to concentrations of 25 mg tissue/mL and samples of pancreatic tissue to concentrations of 11 mg/mL assay buffer. After a centrifugation at 10.000×g for 20 min at 4°C, the pellet was dissolved in 1 mL of *solubilization buffer*, sonicated and subjected to two cycles of freeze/thaw. Following another step of centrifugation (8.000×g, 20 min at 4°C), the supernatant was stored at −80°C until assayed.

MPO activity was measured in 96-well black plates, using 50 μL of sample and 50 μL of *reaction cocktail*. After 60 min of incubation in the dark, fluorescence was recorded using an *Infinite 200 microplate reader* and an excitation/emission wavelength of 550/595 nm, respectively. MPO activity was calculated using a MPO standard delivered with the kit.

### Redox Status in Serum of UCP2^-/-^ and C57BL/6 Mice

To analyze redox status in serum of UCP2^-/-^ and C57BL/6 mice, the ratio of glutathione (GSH) to glutathione disulfide (GSSG) was determined using the EnzyChrom GSH/GSSG Assay according to the manufacturer's instructions (BioAssay Systems, CA, USA). Therefore, each serum sample was divided into two parts. One half was used to measure GSSH, the other to determine total glutathione. In case of GSSG detection, samples were preincubated with 1-methyl-2-vinylpyridinium triflate for 10 min at RT. Afterwards, all samples were deproteinated employing 5% metaphosphoric acid and cleared by centrifugation. The supernatants were mixed with *assay buffer*, incubated with *working reagent* (containing gluthatione reductase and Ellman's reagent), and optical density at 412 nm was determined using an *Infinite 200 microplate reader*. Results are expressed as a ratio of GSH/GSSG.

### *In vitro* Studies with Pancreatic Acini

Acini were freshly prepared from the mouse pancreas by digestion with collagenase [34], [35]; suspended in HEPES (24.5 mmol/L)-buffered medium (pH 7.5) containing NaCl (96 mmol/L), KCl (6 mmol/L), MgCl_2_ (1 mmol/L), NaH_2_PO_4_ (2.5 mmol/L), CaCl_2_ (0.5 mmol/L), glucose (11.5 mmol/L), Na-pyruvate (5 mmol/L), Na-glutamate (5 mmol/L), Na-fumarate (5 mmol/L), minimum essential medium (1% v/v), and bovine serum albumin, fraction V (1% w/v). Immediately after isolation, more than 99.9% of the acinar cells were found to be viable by trypan blue staining. The rate of cell death slightly increased in the course of incubation but still remained below 5% at the latest experimental time point (90 min, see below).

For the detection of protease activities, acini were adjusted to a biovolume concentration of 2 mm^3^/mL living cells. After an equilibration period of 30 min, cerulein (Bachem) was added at the supramaximal concentration of 10 nmol/L for up to 90 min at a temperature of 37°C. Subsequently, acini were washed and resuspended in medium without secretagogue but in the presence of synthetic substrates for trypsin (CBZ-Ile-Pro-Arg)_2_-rhodamine-110 (10 μmol/L) and elastase (CBZ-Ala_4_)_2_-rhodamine-110 (10 μmol/L) (Fisher Scientific, Schwerte, Germany), respectively. To quantify substrate cleavage, cerulein-treated acini and control cultures, together with the substrate, were transferred to 96-well microtiter plates. The change of fluorescence intensity (ΔF) over a time period of 90 min was determined at RT by cytofluorometry at 485 nm excitation and 530 nm emission wavelengths (CytoFluor 2350; Millipore, Bedford, MA) and expressed as ΔF/Δt ratio as previously reported [36].

To measure intraacinar levels of trypsinogen and proelastase, both proenzymes were activated *in vitro* by incubation with active trypsin (which was chosen since enterokinase would cleave the elastase substrate). Therefore, adjusted biovolumes of acinar cells (1.2–1.3 mm^3^/mL) were washed and resuspended in acetate buffer (50 mmol/L, pH 5.0) supplemented with NaCl (100 mmol/L), ethylenediaminetetraacetic acid (4 mmol/L) and dithiothreitol (8 mmol/L). After cell lysis by sonication, the lysates were cleared by centrifugation and the supernatants incubated with trypsin (1 nmol/L; porcine protein; SERVA, Heidelberg, Germany) for 30 min at 37°C. Afterwards, measurements of trypsin and elastase activities were performed as described above, except of that the time period was reduced to 60 min. Subsequently, ΔF/Δt ratios were calculated and corrected by subtracting the corresponding values obtained from control samples where the cell lysate was replaced by assay buffer supplemented with trypsin at 1 nmol/L.

For the detection of intracellular ROS levels, 2′,7′-dichlorofluorescin diacetate (DCFH-DA) was employed. DCFH-DA, a cell-permeable non-fluorescent probe, is de-esterified intracellularly and turns to highly fluorescent 2′,7′-dichlorofluorescein upon oxidation in the presence of hydroxyl, peroxyl and other ROS activity. Therefore, acini were adjusted to a biovolume concentration of 1 mm^3^/mL, transferred to black 96-well plates and preincubated with DCFH-DA (dissolved in dimethyl sulfoxide) at a final concentration of 50 μmol/L for 30 min at RT. Afterwards, the cells were spun down by centrifugation, resuspended in DCFH-DA-free assay medium (HEPES 24.5 mmol/L; pH 7.4, NaCl 96 mmol/L, KCl 6 mmol/L, MgCl_2_ 1 mmol/L, NaH_2_PO_4_ 2.5 mmol/L, glucose 11.5 mmol/L, CaCl_2_ 0.5 mmol/L, Na-pyruvate 5 mmol/L, Na-glutamate 5 mmol/L, Na-fumarate 5 mmol/L, Eagle medium 10% v/v, bovine serum albumin fraction V 1% w/v) and exposed to cerulein at either supramaximal (10 nmol/L) or maximal (0.1 nmol/L) concentrations for 30 min as indicated. Finally, fluorescence intensity was determined at 495 nm excitation and 529 nm emission wavelengths using an automated fluorescence reader (GloMax-Multi+ Detection System, Promega, Madison, WI, USA). Data are expressed as percentage of controls as described in the figure legend.

### Statistical Analysis

Results are expressed as mean ± standard error of the mean (SEM) for the indicated number of animals/samples per experimental protocol. Statistical significance was checked using the Mann-Whitney U test. P<0.05 was considered to be statistically significant.

## Results

### Effects of UCP2 Gene Knockout on the Redox Status

**Figure 1:**
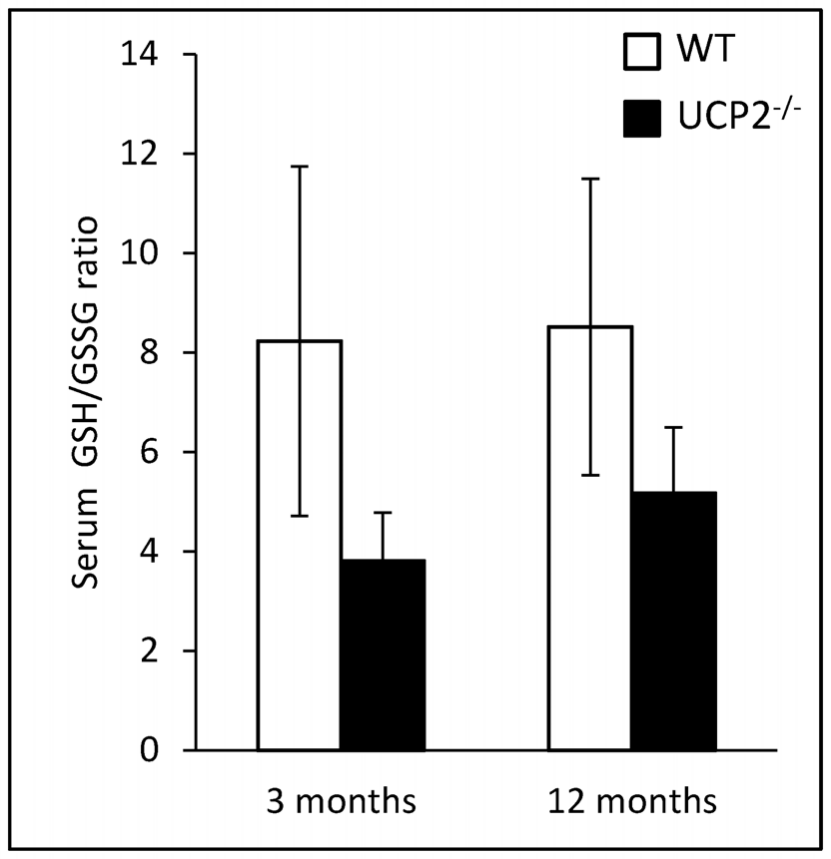
Analysis of redox status in serum of WT and UCP2^-/-^ mice. Serum samples of 3 and 12 months old UCP2^-/-^ and WT C57BL/6 mice (n = 7–8) were used to measure total GSH and GSSG levels. Data are given as GSH/GSSG ratio (mean ± SEM).

In this study, the ratio of reduced and oxidized glutathione (GSH/GSSG) in serum was determined as a surrogate marker of the redox status. Both at an age of 3 and 12 months, UCP2^-/-^ mice displayed the lower ratio, suggesting increased oxidative stress (inverse relationship), although the difference did not reach statistical significance (Fig. 1). In both strains, the GSH/GSSG ratios at 3 and 12 months showed little variation.

### Severity of Pancreatitis in WT and UCP2^-/-^ Mice

**Figure 2:**
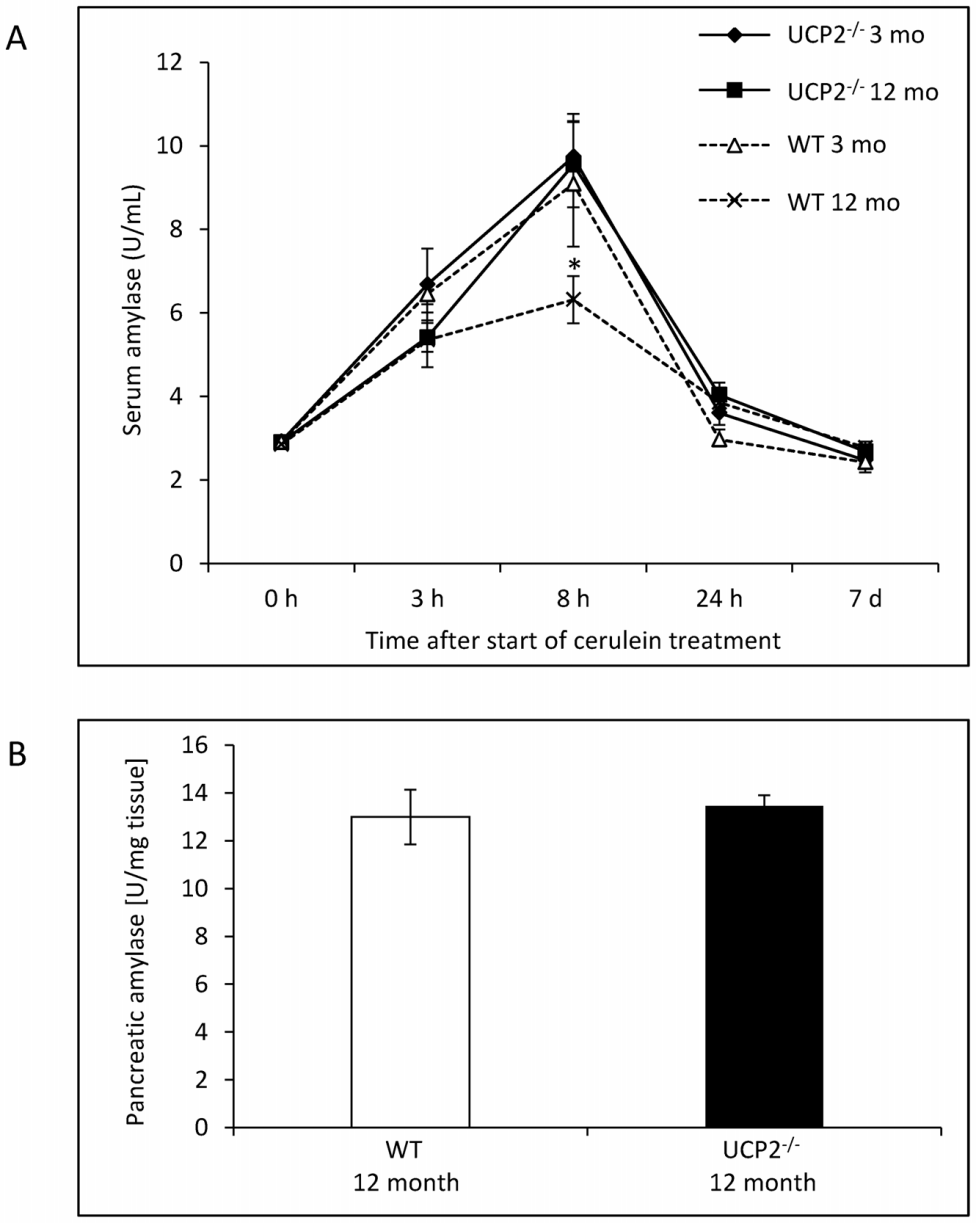
Measurements of α-amylase activities in WT and UCP2^-/-^ mice. (A) Mice of two age groups (3 and 12 months, respectively) were sacrificed at the indicated time points after the start of cerulein treatment (n = 6–8 mice per experimental time point) and serum samples subjected to the analysis of α-amylase activities. Data are expressed as units per milliliter of serum (mean values ± SEM). *P<0.05 (12 months old WT versus UCP2^-/-^ mice). (B) Pancreatic tissue of untreated WT and UCP2^-/-^ mice at an age of 12 months (n = 6 per group) was subjected to the detection of α-amylase activities. Data are expressed as U/mg wet weight (mean ± SEM).

To study the effects of a UCP2 gene knockout on the course of acute experimental pancreatitis, we employed the standard model of 7 repeated cerulein injections at hourly intervals [29], [30]. Again, two age groups, 3 and 12 months old mice, were included in order to determine age-dependent effects of UCP2-deficiency. As shown in Fig. 2 A, mice of both strains and age groups displayed a strong increase of α-amylase levels in serum with a peak after 8 hours and a return to baseline levels until day 7. While α-amylase activities in 3 months old mice of both strains and in 12 months old UCP2^-/-^ mice were very similar at any time point, WT mice at an age of 12 months reached a significantly lower maximum serum enzyme level. Since basal α-amylase activities in pancreatic tissue were the same in 12 months old mice of both strains (Fig. 2 B), the observed difference in maximum serum amylase activity cannot be attributed to a strain-dependent pancreatic content of the enzyme.

**Figure 3:**
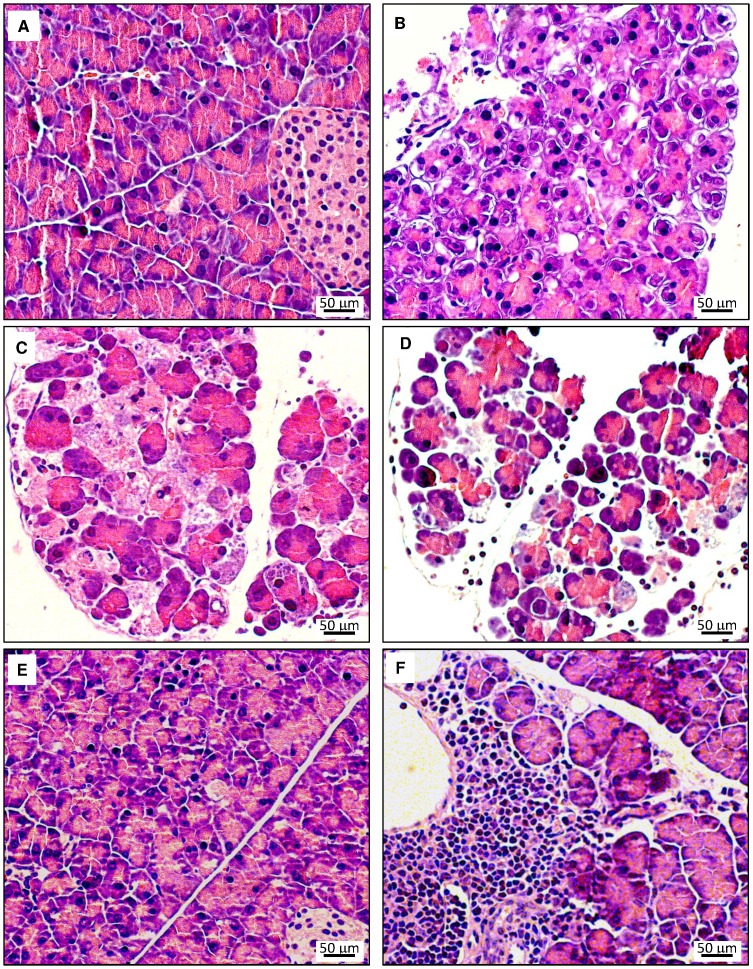
Histopathology of pancreatic lesions in cerulein-treated 12 months old WT C57BL/6 mice. Pancreatic sections were stained with H&E. Photographs A–E display representative examples of pancreatic damage at the time points 0 h, 3 h, 8 h, 24 h and 7 days after the start of cerulein treatment. (A) 0 h – healthy pancreas (B) 3 h – focal cell swelling, vacuolization and cystic degeneration, death of single cells (C) 8 h – in addition to the changes at 3 h progressive interstitial edema and widespread cell death (D) 24 h – interstitial edema, cell degeneration and multiple apoptotic and necrotic cells; presence of interstitial inflammatory cells (E) day 7 – slight residual cell swelling. Photograph (F) from a mouse on day 7 shows an additional incidental finding occasionally observed especially in 12 months old mice: lymphoplasmacytic and eosinophilic interstitial infiltrate surrounding ducts and vessels; compatible with an autoimmune pancreatitis.

**Figure 4:**
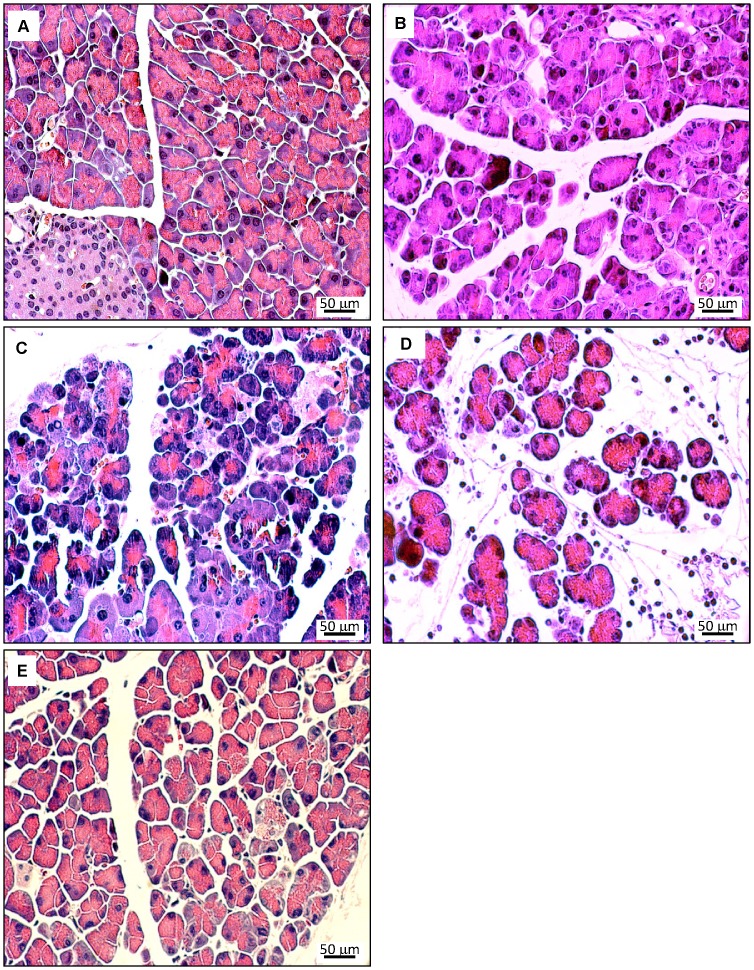
Histopathology of pancreatic lesions in cerulein-treated 12 months old UCP2^-/-^ mice. Pancreatic sections were stained with H&E. Photographs A–E display representative examples of pancreatic damage at the time points 0 h, 3 h, 8 h, 24 h and 7 days after the start of cerulein treatment. In addition to the findings described in Fig. 3, more pronounced histopathological changes at the time points 24 h (D – edema and presence of interstitial inflammatory cells) and day 7 (E – edema and cell damage) were observed.

To further assess pancreatic damage, histological evaluations were performed. In Fig. 3 (A–E), typical pathomorphological changes in the course of acute cerulein-induced pancreatitis are shown for the example of 12 months old WT mice. The characteristic findings include (1) interstitial edema, (2) cell swelling, vacuolization and cystic degeneration, (3) cell death by apoptosis and necrosis and (4) tissue infiltration with inflammatory cells, and reached a maximum after 8–24 h. Until day 7, an almost complete *restitutio ad integrum* was observed (for further details, see legend to Fig. 3). As an additional finding (which is agreement with previous studies [37]), large and compact infiltrates of immune cells, surrounding ducts and vessels, were occasionally detected in 12 months old mice and in exceptional cases also in 3 months old mice of both strains. The latter type of inflammation, which is compatible with a chronic autoimmune pancreatitis (AIP) in mice [38], was independent of cerulein injections (data not shown) and therefore not considered for scoring. For comparison, Fig. 4 shows histopathological changes in age-matched UCP2^-/-^ mice. While the principal findings were identical, a tendency to increased severity at late time points (24 h and 7 d after start of cerulein treatment, Fig. 4 D and E) was observed.

**Figure 5:**
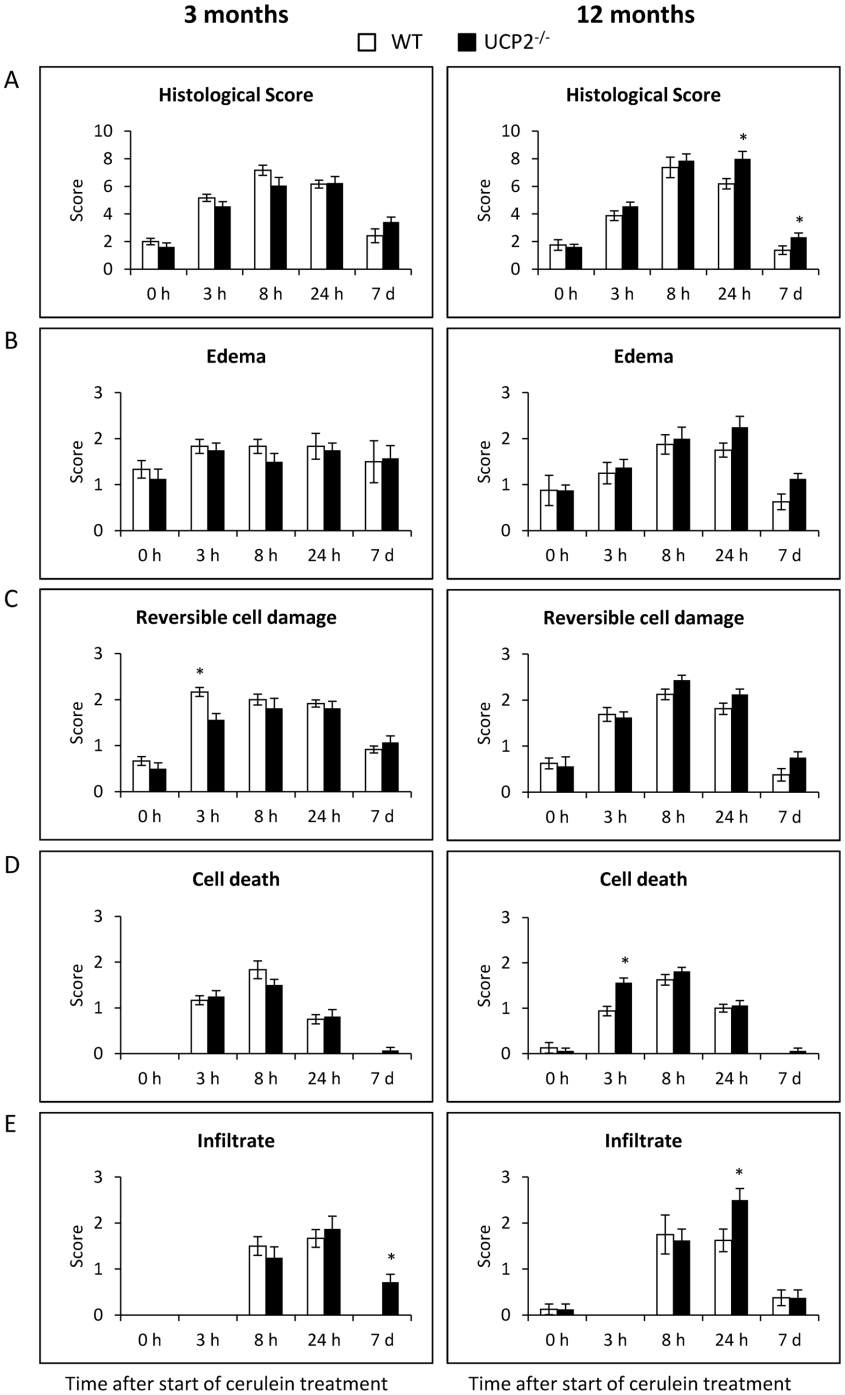
Semiquantitative evaluation of pancreatic lesions in cerulein-treated WT and UCP2^-/-^ mice. Three and 12 months old UCP2^-/-^ and WT C57BL/6 mice were treated with cerulein as indicated (n = 6–8 per time point, strain and age group). Pancreatic sections of the mice were stained with H&E and scored as described in the “Materials and methods” section. The total histological score shown in (A) represents the sum of the scores for edema (B), potentially reversible cell damage (C), cell death (D) and infiltration with immune cells (E), each of which scored on a scale from 0–3. Data are shown as mean ± SEM. *P<0.05 (WT versus corresponding UCP2^-/-^ mice).

For further analysis, histopathological changes were assessed by semiquantitative scores. At an age of 3 months, WT and UCP2^-/-^ mice were virtually indistinguishable with respect to their total histopathological scores at any time point (Fig. 5 A, left panel). This equal disease severity is also reflected by the sub-scores for the individual parameters edema (5 B), potentially reversible cell damage (5 C), cell death (5 D) and presence of inflammatory cells (5 E), which showed no significant differences between the strains (with two non-systematic exceptions as indicated in 5 C and 5 E). In contrast, at an age of 12 months total histopathological scores differed in that UCP2^-/-^ mice were scored significantly higher at the two late time points 24 h and 7 d, suggesting a retarded regeneration process (Fig. 5 A, right panel). In addition to the total scores, also the sub-scores for individual parameters (Fig. 5 B–E) point to a slightly increased disease severity in UCP2^-/-^ mice, although statistically significant differences were rare.

**Figure 6:**
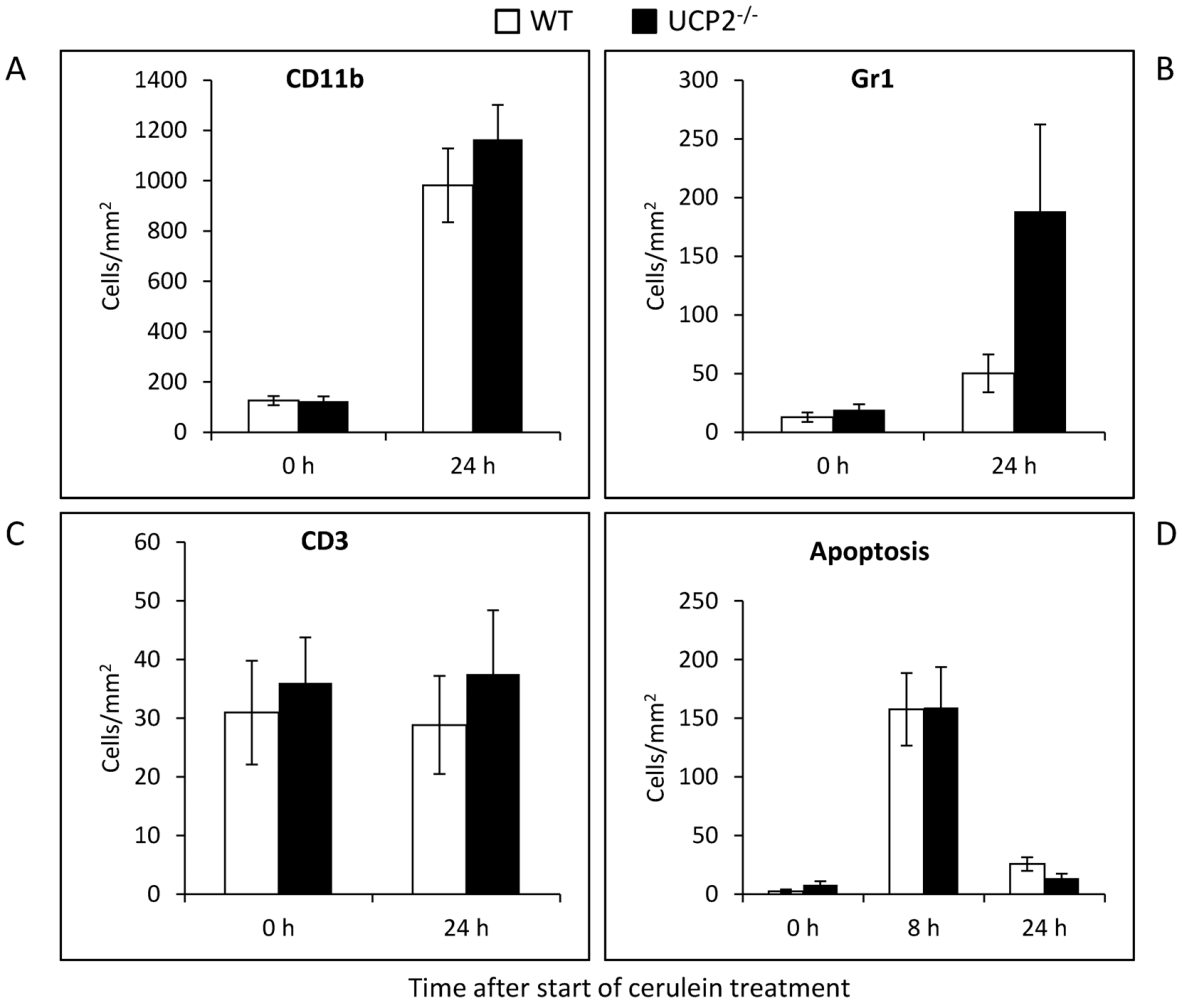
Immunohistochemical analysis of leucocyte infiltrates and quantification of apoptotic cells in cerulein-treated WT and UCP2^-/-^ mice. Pancreatic sections of 12 months old mice at the indicated time points after initiation of cerulein treatment (n = 8 per group) were processed applying the ABC technique and antibodies to CD11b (A), Gr1 (B) and CD3 (C), or stained with the ApopTag Kit for the detection of apoptotic cells (D). Positive-stained cells were counted, and mean values ± SEM were calculated. *P<0.05 (WT versus corresponding UCP2^-/-^ mice).

A detailed analysis of inflammatory cell infiltrates (outside of AIP-like foci) revealed increased numbers of cells expressing CD11b (monocytes/macrophages, neutrophils, natural killer cells and granulocytes) and Gr1 (neutrophils), but not of CD3-positive lymphocytes, in pancreatic tissue of 12 months old mice of both strains at 24 h after the start of cerulein treatment (Fig. 6A–C ). Furthermore, a tendency to higher numbers of all three types of cells in UCP2^-/-^ mice than in WT animals was observed. The number of apoptotic cells at 0, 8 and 24 h after induction of pancreatitis did not differ between 12 months old mice of the two strains (Fig. 6 D).

**Figure 7:**
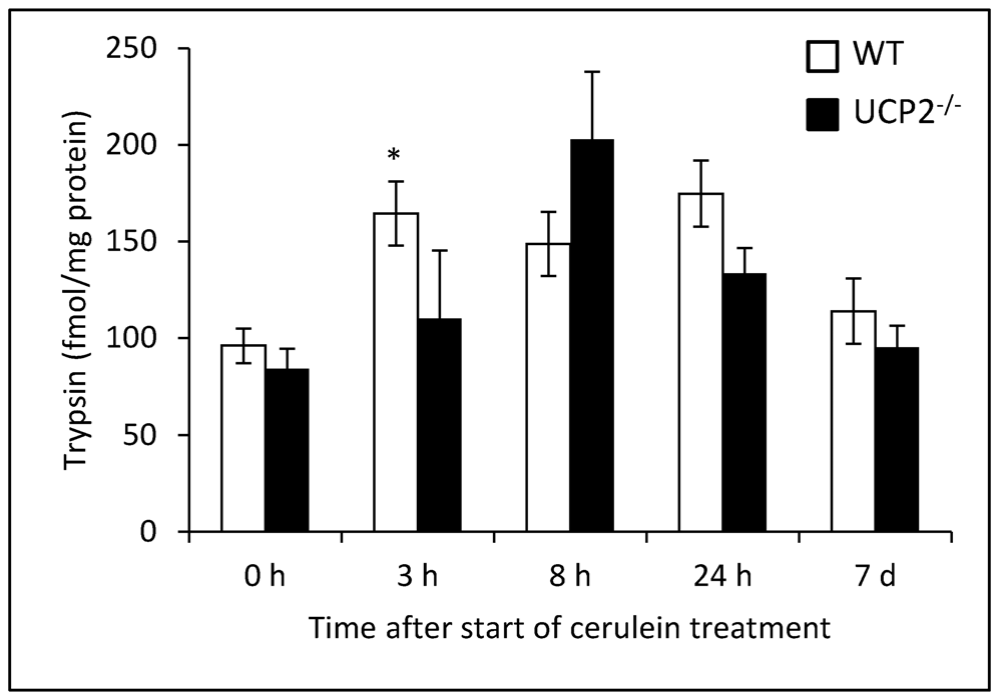
Determination of trypsin activity in pancreatic tissue of cerulein-treated WT and UCP2^-/-^ mice. Tissue samples of 12 months old mice at the indicated time points after initiation of cerulein treatment (n = 7–8 per group) were subjected to the quantification of trypsin activity as described in the “Materials and methods” section. Data are expressed as fmol active trypsin per mg pancreatic protein (mean ± SEM). *P<0.05 (WT versus corresponding UCP2^-/-^ mice).

We also determined levels of active trypsin in pancreatic tissue of WT and UCP2^-/-^ mice. As expected, in both strains a transient increase of trypsin activity in the course of experimental pancreatitis was observed. Although the highest absolute value was detected in UCP2^-/-^ mice (8 h after the first cerulein injection), there was not a general tendency to higher levels in the knockout strain. On the contrary, 3 h after the induction of pancreatitis, pancreatic trypsin activity was significantly higher in WT mice (Fig. 7).

### *In vitro* Studies with Pancreatic Acini

**Figure 8:**
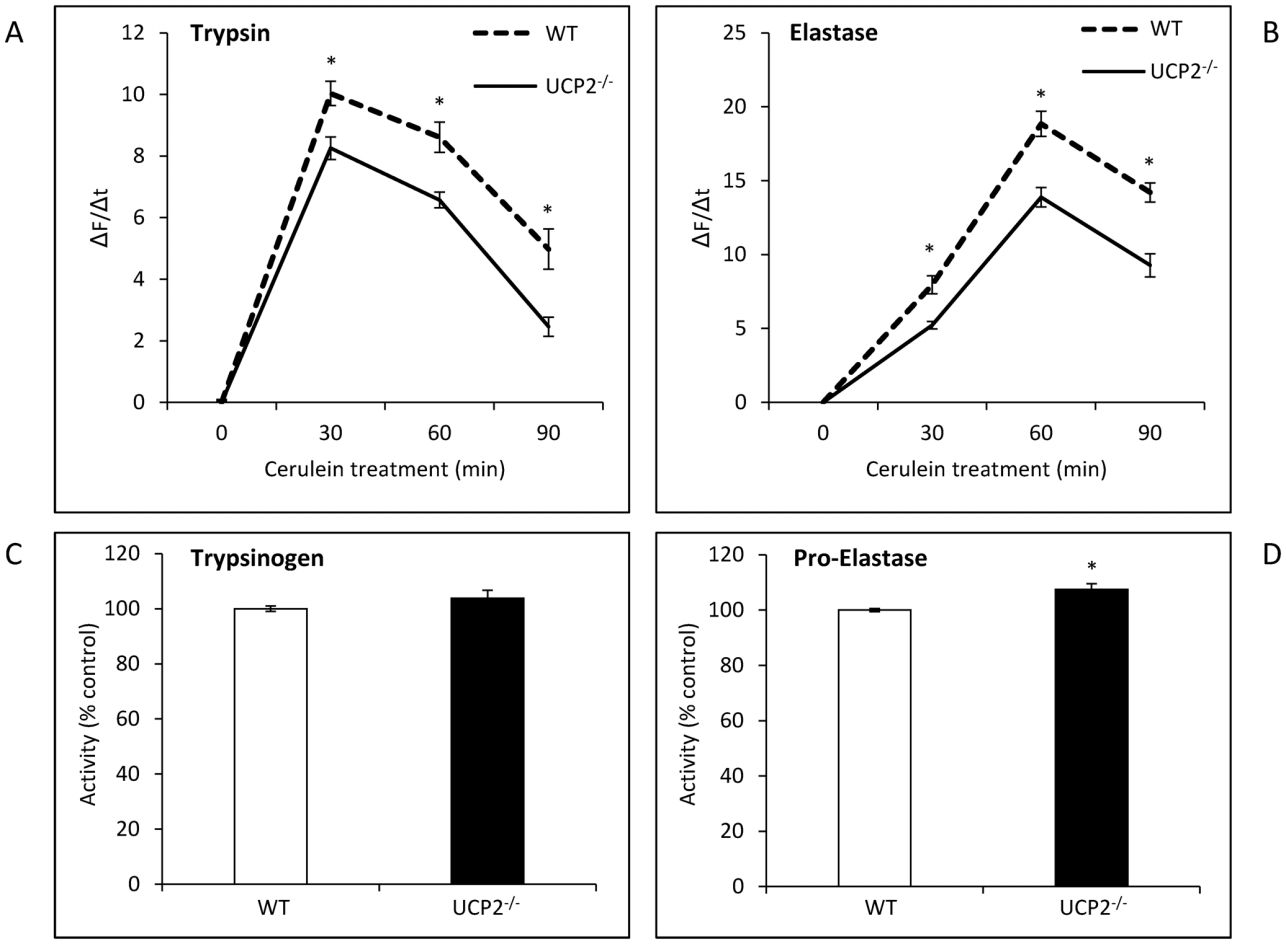
Trypsin and elastase activities in pancreatic acini isolated from WT and UCP2^-/-^ mice. Isolated pancreatic acini of 12 months old WT and UCP2^-/-^ mice were treated *in vitro* with cerulein (10 nmol/L) for up to 90 min. Subsequently, cleavage of rhodamine 110-labeled synthetic substrates for (A) trypsin and (B) elastase was recorded by cytofluorometry. To compare proteolytic activities, ΔF/Δt ratios were calculated and data expressed as (ΔF/Δt)_cerulein_ - (ΔF/Δt)_untreated_ (mean ± SEM, n = 36). *P<0.05 (WT versus corresponding UCP2^-/-^ mice). The intracellular levels of trypsinogen (C) and proelastase (D) were assessed by incubating the lysates obtained from unstimulated WT- and UCP2^-/-^ cells of 12 months old mice with active trypsin as described in the “Materials and methods” section. A value of 100 percent corresponds to the averaged activity of *in vitro*-activated trypsin from WT cells. Data are shown as mean ± SEM (n = 18). *P<0.05 (WT versus corresponding UCP2^-/-^ mice).

Using isolated pancreatic acini from 12 months old mice, we studied intracellular activation of pancreatic proteases in response to a treatment with cerulein *in vitro* (Fig. 8). As expected, a rapid and transient increase of both trypsin (8 A) and elastase (8 B) activities was observed. Interestingly, activation of both proteases was significantly stronger in the WT strain than in UCP2^-/-^ mice. In contrast, the intracellular level of trypsinogen did not differ between the two strains while proelastase concentration was even slightly higher in acinar cells of UCP2^-/-^ mice (8 C and D). Thus, the higher activities of trypsin and elastase in cerulein-treated WT cells do not result from an increased expression of the proenzymes.

**Figure 9:**
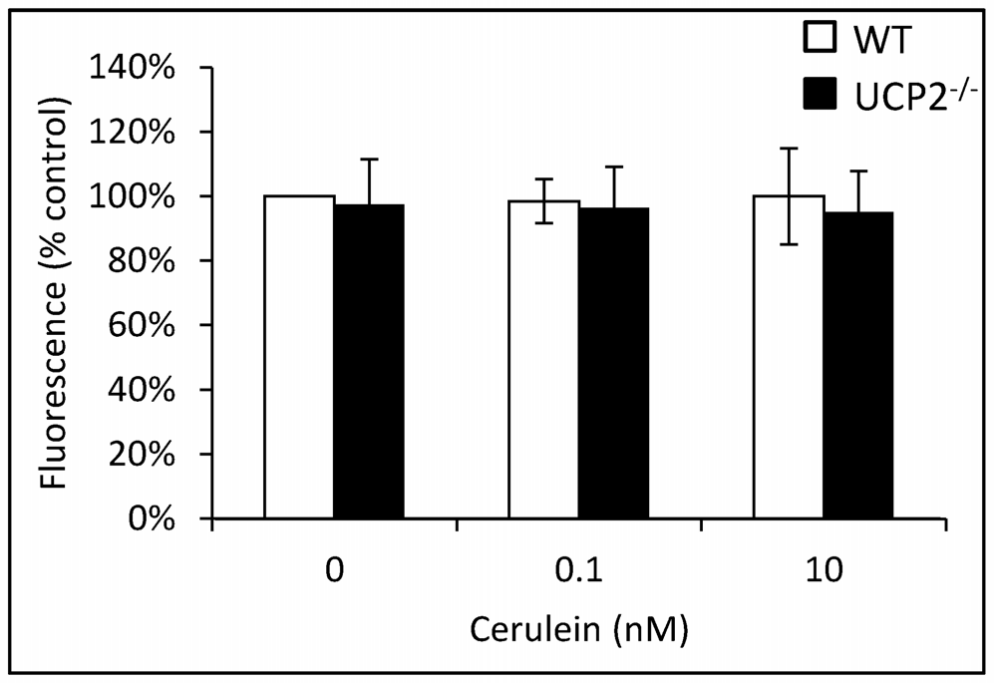
ROS levels in pancreatic acini isolated from WT and UCP2^-/-^ mice. DCFH-DA-labeled pancreatic acini of 12 months old WT and UCP2^-/-^ mice were treated *in vitro* with cerulein at the indicated concentrations for 30 min before fluorescence intensity of 2′,7′-dichlorofluorescein was recorded. Data of 6 independent samples per experimental protocol were used to calculate mean values ± SEM. *P<0.05 (WT versus corresponding UCP2^-/-^ mice).

Furthermore, there was no difference in the intraacinar ROS levels of both strains, independent of the presence or absence of cerulein (which also displayed no effect at the two concentrations tested) (Fig. 9). Similar results as presented in Fig. 8 and 9 were also obtained for 3 months old mice (data not shown). Together, these data point to effective mechanisms of antioxidative defense in pancreatic acinar cells and suggest that the increased severity of cerulein pancreatitis in UCP2^-/-^ mice (Fig. 5) is unrelated to the investigated acinar cell functions.

### MPO Activity in Lung and Pancreatic Tissue of Cerulein-Treated Mice

**Figure 10:**
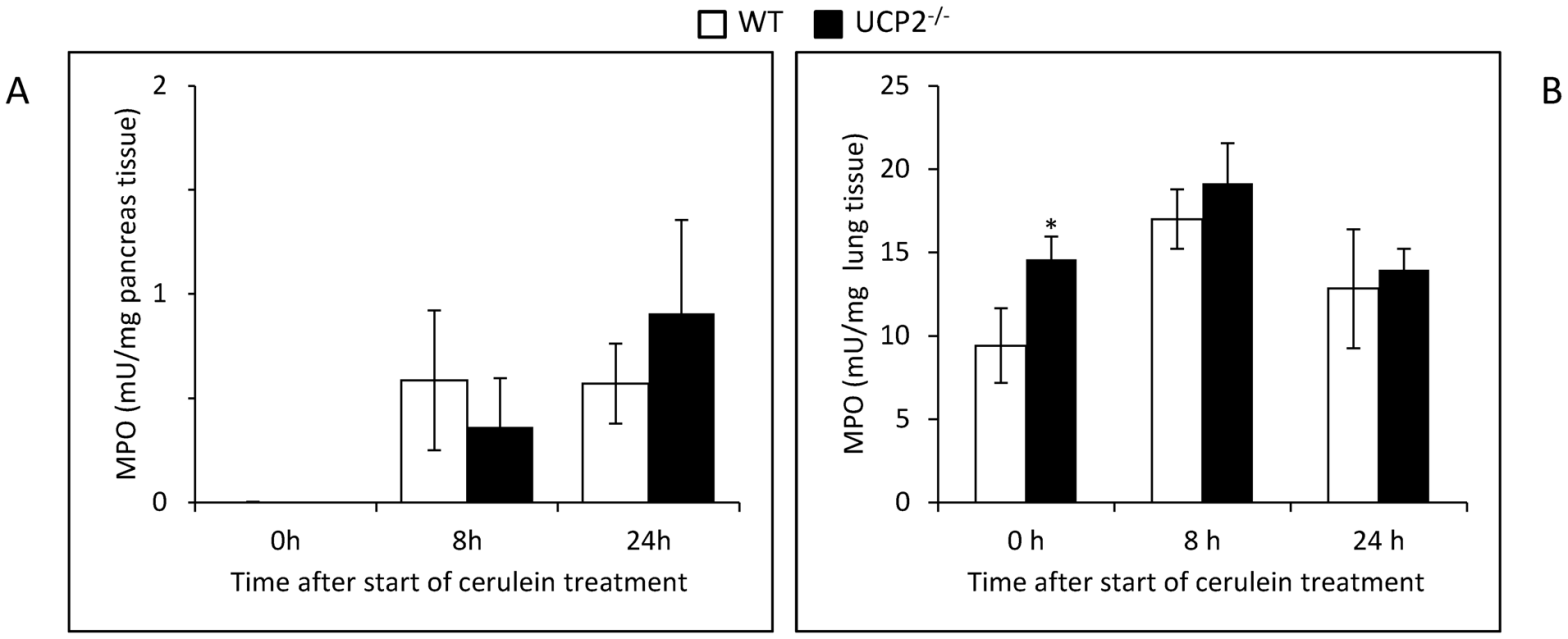
MPO activities in pancreatic and lung tissue of cerulein-treated WT and UCP2^-/-^ mice. Tissue samples (A, pancreas; B, lung) of 12 months old mice at the indicated time points after initiation of cerulein treatment (n = 8 per group) were subjected to the quantification of MPO activity as described in the “Materials and methods” section. Data are expressed as mU/mg wet weight (mean ± SEM). *P<0.05 (WT versus corresponding UCP2^-/-^ mice).

Given that (1) cerulein pancreatitis is associated with intrapancreatic accumulation of leucocytes and (2) UCP2^-/-^ deficiency has been linked to dysfunction of monocytes/macrophages [26], we reasoned that tissue activities of MPO might vary between the two strains. In pancreatic tissue of 12 months old mice, the highest absolute value was measured in the knockout strain (24 h after induction of pancreatitis; Fig. 10), although the differences were not statistically significant. In lung tissue, MPO activities were consistently higher in UCP2^-/-^ mice than in control animals (0, 8 and 24 h after the first cerulein injection, respectively), with a statistically significant difference for the basal activity.

## Discussion

The results of this study show that UCP2^-/-^ mice at an advanced age of 12 months display an increased susceptibility to cerulein-induced experimental AP. The effect of UCP2 deficiency on the severity of the disease was altogether moderate, with a more sustained pancreatic damage (indicated by the histopathological score; Fig. 5A) as the main finding. Furthermore, a stronger increase of α-amylase activity was observed in the knockout strain.

UCP2 functions as a negative regulator of mitochondrial ROS production and may exert cytoprotective effects [17]–[21]. Since pancreatic acinar cells express UCP2 [16], we reasoned that acini derived from UCP2-deficient mice might show relevant functional defects. Intraacinar ROS levels, however, were found to be not increased, suggesting that antioxidant defense of pancreatic acinar cells is efficient enough to compensate for the loss of the uncoupling protein. Interestingly, premature activation of the proteases trypsin and elastase was even attenuated in cells from the knockout strain. This phenomenon might reflect an adaptive mechanism of the cells and needs to be studied further at the molecular level. In agreement with the results of the *in vitro* experiments, the *in vivo* studies showed that cerulein-treated UCP2^-/-^ mice had at least no higher intrapancreatic levels of active trypsin than WT animals. Together, both approaches argue against the possibility that functional defects of pancreatic acinar cells are responsible for the increased severity of cerulein pancreatitis in 12 months old UCP2^-/-^ mice.

Previous studies have identified infiltrating leucocytes as the most important source of excessive pancreatic ROS production in AP [39], [40]. Here, we observed gradually higher levels of pancreatic MPO activity in the knockout strain. Considering that macrophages of UCP2^-/-^ mice display a higher oxidative burst compared to WT macrophages [26], [41], it seems likely that the increased pancreatic damage in the knockout strain is caused, at least in part, by a ROS-mediated increased activity of infiltrating leucocytes. Specific mechanisms of UCP2 action in this context may also include regulatory effects on immune cell migration and the production of cytokines and nitric oxide, as previously demonstrated in models of infection, inflammation and autoimmunity [26]. Of note, increased levels of MPO activity were also observed in the lungs of cerulein-treated (and untreated) UCP2^-/-^ mice, suggesting a systemic impact of the knockout in our experimental model.

In accordance with previous studies [25], we found that the ratio of GSH/GSSG in serum was lower in UCP2^-/-^ mice of different age groups than in WT animals (although not to the same extent as previously reported), suggesting diminished antioxidative resources throughout the lifespan of the animals. Nevertheless, in young adult mice (3 months old) no systematic effects of UCP2-deficiency on the course of experimental AP were observed. Age-dependent effects of UCP2 have also been detected in other experimental disease models [25] and may reflect an increasing importance of the uncoupling protein in ageing individuals in the context of an increased oxidative stress burden. We therefore hypothesize that the efficiency of adaptation to a lack of UCP2 expression decreases with the age of the animals.

The precise role of ROS in the pathogenesis of AP is a matter of ongoing debate. In summary, our results underscore the important function of ROS-producing inflammatory cells in the progression of the experimental AP and suggest inflammatory cells as a specific target for future studies with antioxidants.
